# Effect of photocatalytic pretreatment on the biodegradation of n-hexane vapours in a biofilter

**DOI:** 10.1016/j.mex.2020.100991

**Published:** 2020-07-10

**Authors:** Yaghoub Hajizadeh, Negar Jafari, Mehdi Mokhtari, Amir Mohammadi, Seyed Mojtaba Momtaz, Farzad Fanaei, Ali Abdolahnejad

**Affiliations:** a Department of Environmental Health Engineering, Faculty of Health, Environmental Research Center, Research Institute for Primordial Prevention of Non-Communicable Disease, Isfahan University of Medical Sciences, Isfahan, Iran; b Environmental Science and Technology Research Center, Department of Environmental Health Engineering, Faculty of Health, Shahid Sadoughi University of Medical Sciences, Yazd, Iran; c Department of Public Health, Maragheh University of Medical Sciences, Maragheh, Iran; d Department of Environmental Health Engineering, Faculty of Health, Bam University of Medical Sciences, Bam, Iran; e Department of Environmental Health Engineering, Faculty of Health, Iran University of Medical Sciences, Tehran, Iran

**Keywords:** Photoreactor, Biofilter, Hybrid system, n-hexane vapor

## Abstract

Hydrophobic volatile organic compounds (VOCs) such as n-hexane are not completely biodegradable by a single biofilter. So, in the present study, a photoreactor system packed with scoria granules coated with TiO_2_, as a pretreatment unit, was used for increasing the removal efficiency of n-hexane by a biofilter during an operation period of 191 days. The inlet and outlet concentration of n-hexane was analyzed with a gas chromatography coupled with a flame ionization detector (GC/FID). The results indicated that the removal efficiency of the single biofilter with input concentrations of 0.18 – 1 g/m^3^ at empty bed residence times (EBRTs) of 30, 60, and 120 s was 10.06%, 21.45%, and 46.8%, respectively. When the photoreactor was used as a pretreatment system, the removal efficiency of the combined system in corresponding EBRTs was improved to 39.79%, 63.08%, and 92.60%, respectively. The results proved that the combined system provided higher removal efficiencies than the single biofilter. Thus, the application of the photoreactor as a pretreatment step was much effective in increasing the removal efficiency of n-hexane from the polluted air by the biofilter.

## Graphical abstract

**Figure:**
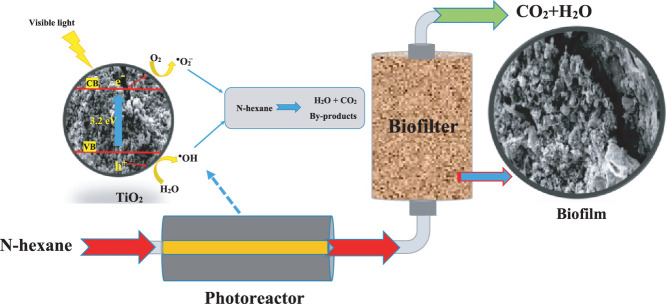

Specifications table

**Table:** 

Subject Area	Environmental Science
More specific subject area	Air pollution control
Protocol name	**Effect of photocatalytic pretreatment on the biodegradation of n-hexane vapours in a biofilter**
Reagents/tools	GC/FID, model: Varian CP-3800, USA, column characteristics: type: CP-sil5 CB, column: 15.0 *m* × 0.25 mm × 0.45 μm, injector characteristics: the injector temperature 200 °C, Nitrogen (N_2_) with a flow rate of 1.2 and 25 ml/min was as the carrier gas and make-up gas, respectively. CO_2_ analyzer AZ Model 77,535 (Taiwan).
Experimental design	*All sampling and analysis of n-hexane concentration were conducted according to previous studies*[1], [2]*.*
Trial registration	*–*
Ethics	*–*
**Value of the Protocol**	• *N-Hexane, as a hydrophobic VOCs is widely used as a cleaning agent in textiles, furniture, leather products and as a solvent in oil and grease removal and oil extraction of vegetables**[3**,**4]**.* • *N-hexane due to its low bioavailability and low solubility in water is poorly removed in biological treatment systems*[5], [6], [7]*.* • *Thus, the photochemical oxidation process (PCO) as a pretreatment unit can decompose variety of HOCs by producing oxidizing species such as OH^·^ radicals and ozone, and convert them to biodegradable compounds*[8], [9], [10]*.* • *So that, the photoreactor by reducing the toxicity of n-hexane and increasing its bioavailability can enhance its removal efficiency in the biofilter as a combined system (photoreactor-biofilter)**[11]**.*

## Description of protocol

### Pilot experimental set-up and operation

**Fig. 1:**
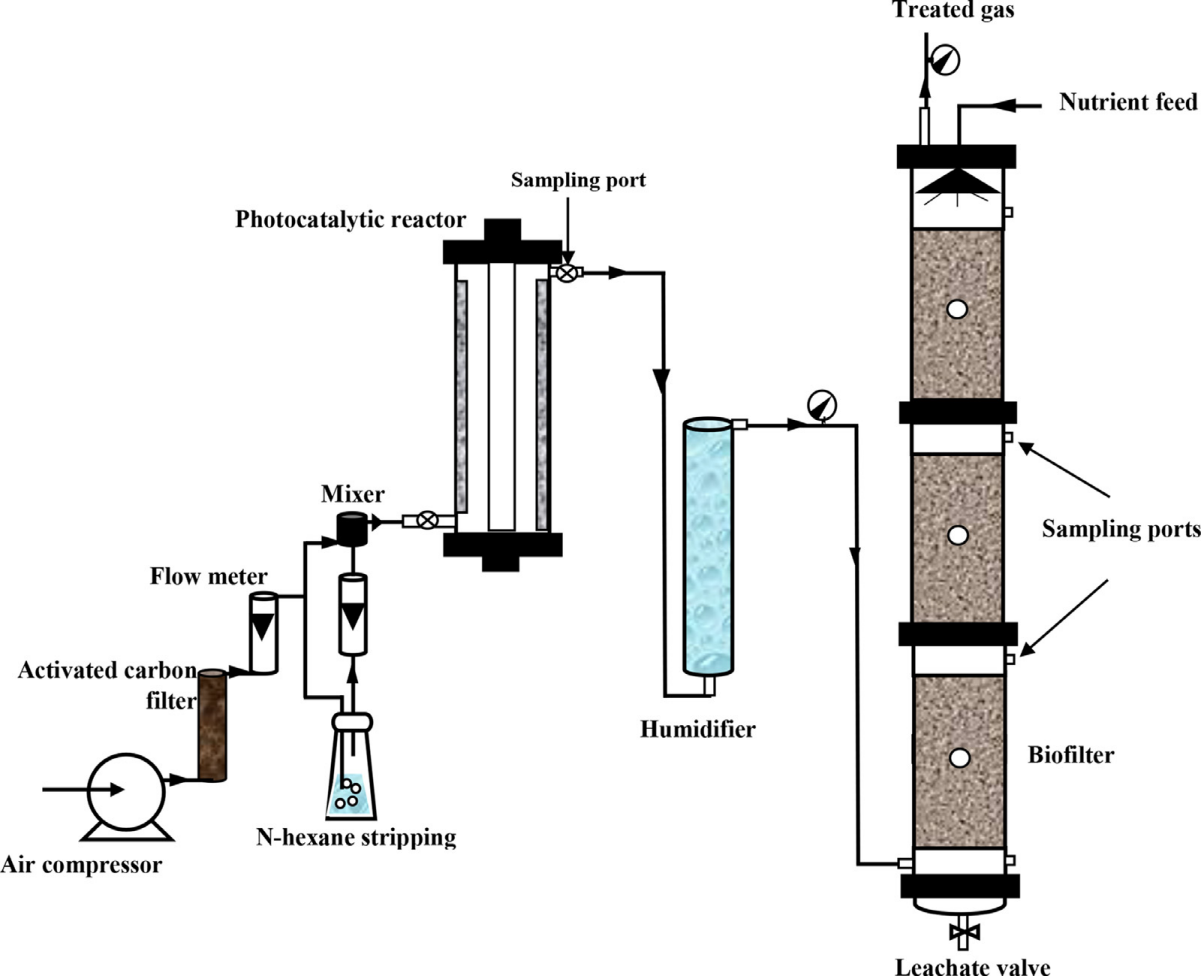
Schematic of the laboratory scale combined system (Photoreactor-biofilter).

The photocatalytic reactor was comprised of a stainless steel tube with 50 cm height and 10 cm internal diameter, and an effective volume of 3.6 L. A low-pressure mercury UV lamp (254 nm) was placed in the photoreactor center. The space between the quartz tube and stainless steel tube was filled with TiO_2_/Scoria catalysts. Scoria with a specific surface area of ​​0.67 m^2^/g, 3 to 5 mm in diameter and porosity of 60% was used as a support for TiO_2_ coating. The multilayers biofilter was made of a stainless steel column with a height, internal diameter, and an effective volume of 120 cm, 15 cm and 15.8 L, respectively. The schematic of the combined system (photoreactor-biofilter) is shown in Fig 1. More details are available in our previous study [11]. After the start-up step, the performance of the combined system was surveyed in the operation period of 191 days (Table 1).

**Table 1:** Operational conditions of the single biofilter and the combined system (photoreactor-biofilter).

Phase	Operation days	EBRT (s)	Inlet concentration range (gm^3^)	Inlet loading range (gm^−3^h^−1^)	Use of Photocatalytic reactor
*Start-up*	1–30	120	0.18 – 0.43	5.4 – 12.9	NO
*I*	31–55	120	0.19 - 0.91	5.7 – 27.3	NO
*II*	56–78	60	0.23 – 1.00	13.8 – 60.0	NO
*III*	79–101	30	0.2 – 0.87	24 – 104.4	NO
*IV*	102–131	120	0.17 – 0.97	5.1 – 29.1	YES
*V*	132–161	60	0.11 – 0.84	6.7 – 51.5	YES
*VI*	162–191	30	0.12 – 0.88	14.3 – 104.9	YES

### Chemicals and preparation of TiO_2_/scoria catalyst

All chemicals for preparation of the nutrient solution and TiO_2_ nano-powder (TiO_2_, anatase, ≥99%) were prepared from Merck Company. TiO_2_ coating was performed according to the follow procedures:

(1) 10 g of TiO_2_ powder was added to 150 mL of ethanol and its pH was adjusted at 3.5 HNO_3_ (0.5 N).
(2) The resulting slurry was entirely diffused with an ultrasound bath (15 min).
(3) For coating, the TiO_2_ on the scoria granules, 45 g of these granules were shed to the slurry and slowly blended for 1 h to provide better adsorption of the TiO_2_ onto the granules.
(4) The slurry solution was evaporated at 80 °C.
(5) The scoria granules were calcinated at 450 °C in a laboratory furnace for 30 min.

Finally, for removing the catalyst which is not attached properly to the scoria granules, the granules washed 2 times with distilled water and air bubbling for a few minutes. More details are available in our previous study [11].

### Analytical method

The inlet and outlet concentrations of n-hexane in the combined system (Photoreactor-Biofilter) were measured by gas chromatography (GC/FID, Varian CP-3800, USA) equipped with an FID detector according to the previous study [1]. For evaluation of n-hexane concentrations, 0.5 mL of inlet and outlet polluted gas was sampled by gas-tight syringe and injected directly into the GC-FID. To evaluate the removal efficiency (REs) of n-hexane, the overall more 1000 samples were taken from the combined system during the operation period so that from each sampling point, two samples were derived daily and the average values were presented in g/m^3^.

### Effect of photocatalytic pretreatment on the performance of the biofilter

**Fig. 2:**
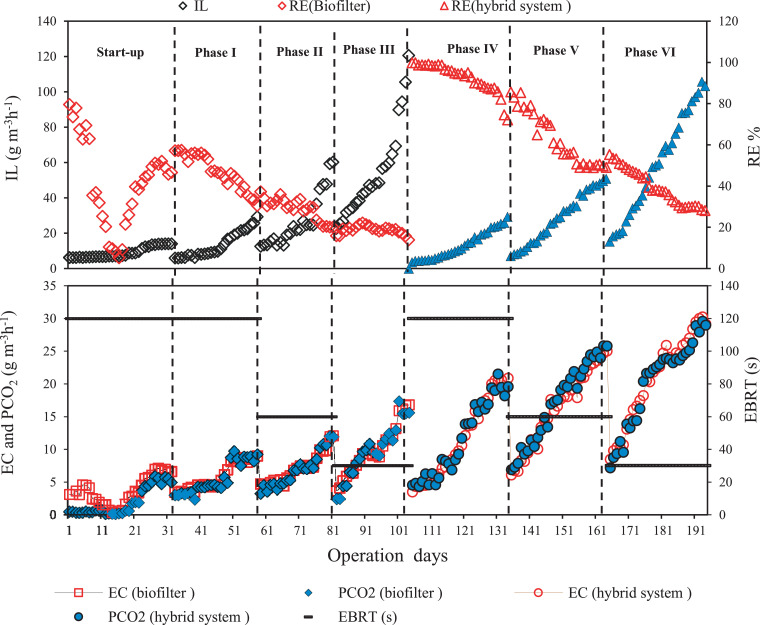
Effect of photocatalytic pretreatment on n-hexane RE, EC and PCO_2_ vs. IL in single biofilter and combined system (Photoreactor-biofilter).

The changes of RE and elimination capacity (EC) of n-hexane with and without inclusion of the photoreactor as a pretreatment unit in various inlet loading (IL) were presented in Fig. 2. The average removal efficiency of n-hexane in the sole biofilter with input concentrations of 0.18 – 1 g/m^3^ at EBRTs of 30, 60, and 120 s was 10.06%, 21.45%, and 46.8%, respectively. This low removal efficiency may be related to the low water solubility and high Henry's coefficient of n-hexane. Because n-hexane as a hydrophobic compound is not easily caught by microorganisms for metabolism. So, to enhance the solubility, mass transfer rate and bioavailability of hydrophobic compounds in the biofilter bed, utilization of a combined system like AOP–biofilter is a good selection [11]. Therefore, when the photoreactor was added as a pretreatment system, the removal efficiency of the combined system in corresponding EBRTs was improved to 39.79%, 63.08%, and 92.60%, respectively. This displays that the photoreactor has a synergistic effect on the removal of n-hexane by the biofilter. Because the TiO_2_ film with the absorption of ultraviolet radiation and production of the free radicals (OH^•^ and O2^•–^) can decompose n-hexane and transform it into CO_2_ and H_2_O and other biodegradable compounds [11,12]. According to the results, the mean removal efficiency of n-hexane by UV photolysis alone (without TiO_2_ catalyst) was less than 5%, which is negligible. Because of low impinging UV irradiation and low gas residence time in the photoreactor, UV photolysis alone was not efficient in mineralization of n-hexane. Also, the results of the present study showed that the maximum removal efficiency of n-hexane in the combined system and in the single biofilter occurred in EBRTs of 120 s. Because higher EBRT due to adequate contact time for pollutant transfer from the gas phase to the biofilm as well as the capacity of the microbes to capture, adsorb and degrade the pollutants, usually leads to higher removal efficiency [13].

According to Fig. 2, a linear positive correlation exists between carbon dioxide production (PCO_2_) and the EC of n-hexane in the single biofilter and combined system. But, the increasing trend of EC against PCO_2_ and CO_2_ production rate in the combined system was more than the single biofilter. This indicates that the mineralization rate of n-hexane due to the presence of a photoreactor as a pretreatment system in the combined system was higher than the single biofilter [8,11]. In our study, 2-hexanone, 3-hexanone and 3-Methyl-butanal were identified as by-products of n-hexane in the output of the photoreactor. The solubility of 2-hexanone (14 g/L) and 3-hexanone (7 g/L) in water is much more than n-hexane (9.5 mg/L). 3-Methyl-butanal, as an aldehyde, is much less toxic than n-hexane. So, compared to n-hexane, they are readily available to microorganisms in the biofilter bed. However, a distinct combination of the peaks with Mass Spectral Library by high match quality was not detected in the output of the biofilter. This could be due to the production of free radicals (OH• and O2•–) in the photoreactor, and thereby breaking down and converting organic compounds into the decomposable intermediate products which were completely removed in the biofilter bed [13].

## Conclusions

N-hexane due to its low water solubility and low bioavailability is not properly removed by the single biofilter. So, in the present study, a photoreactor packed with scoria granules coated with TiO_2_, as a pretreatment unit, was used to increase the removal efficiency of n-hexane by a biofilter during an operation period of 191 days. The results showed that the average removal efficiency of n-hexane in the single biofilter with input concentrations of 0.18 – 1 g/m^3^ in EBRTs of 30, 60, and 120 s was 10.06%, 21.45%, and 46.8%, respectively. Whereas, with the inclusion of the photoreactor before the biofilter, the removal efficiency of the combined system was increased to 39.79%, 63.08%, and 92.60%, respectively in corresponding EBRTs. Because in the photoreactor, TiO_2_ with the absorption of UV radiation and production of the free radicals can decompose n-hexane and transform it to biodegradable materials. Also, the mean removal percentage of n-hexane by UV photolysis alone (without TiO_2_ catalyst) was less than 5%, which is negligible. The maximum removal efficiency of n-hexane in the combined system and in the single biofilter was observed in EBRT of 120 s. 2-hexanone, 3-hexanone and 3-Methyl-butanal were identified as by-products of n-hexane in the output of the photoreactor. Between the by-products, the water solubility of 2-hexanone, and 3-hexanone is more than n-hexane and, the toxicity of 3-methyl-butanal is less than n-hexane. So, compared to n-hexane, they are readily available to microorganisms in the biofilter bed for biodegradation. While no distinct peaks of by-products with Mass Spectral Library by high match quality were detected in the output of the biofilter. Finally, the results of our study demonstrated that using a photoreactor as a pretreatment before biofilter can enhance the removal efficiency of n-hexane.

## Declaration of Competing Interest

None.

## References

[1] Mokhtari M., Hajizadeh Y., Ebrahimi A.A., Shahi M.A., Jafari N., Abdolahnejad A. (2019). Enhanced biodegradation of n-hexane from the air stream using rhamnolipid in a biofilter packed with a mixture of compost, scoria, sugar beet pulp and poplar tree skin. Atmos. Pollut. Res.

[2] Mokhtari M., Hajizadeh Y., Jafari N., Ebrahimi A.A., Abdolahnejad A. (2019). Improved biodegradation of hydrophobic volatile organic compounds from the air stream in a multilayer biofilter. MethodsX.

[3] Bhuiya M.M.K., Rasul M., Khan M., Ashwath N., Mofijur M. (2020). Comparison of oil extraction between screw press and solvent (n-hexane) extraction technique from beauty leaf (Calophyllum inophyllum L.) feedstock. Ind. Crops. Prod..

[4] Li X., Yu T., Wang S., Wang Q., Li M., Liu Z., Xie K. (2020). Diallyl sulfide-induced attenuation of n-hexane-induced peripheral nerve impairment is associated with metabolic inhibition of n-hexane. Food Chem. Toxicol..

[5] He S., Ni Y., Lu L., Chai Q., Yu T., Shen Z. (2020). Simultaneous degradation of n-hexane and production of biosurfactants by Pseudomonas sp. strain NEE2 isolated from oil-contaminated soils. Chemosphere.

[6] Cheng Y., Li X., Liu H., Yang C., Wu S., Du C., Nie L., Zhong Y. (2020). Effect of presence of hydrophilic volatile organic compounds on removal of hydrophobic n-hexane in biotrickling filters. Chemosphere.

[7] He S., Ni Y., Lu L., Chai Q., T Yu, Shen Z., Liu H., Yang C. (2019). Enhanced biodegradation of n-hexane by Pseudomonas sp. strain NEE2. Sci. Rep.

[8] Zhang J., Hu Y., Qin J., Yang Z., Fu M. (2020). TiO2-UiO-66-NH2 nanocomposites as efficient photocatalysts for the oxidation of VOCs. Chem. Eng. J..

[9] Fernandes A., Gągol M., Makoś P., Khan J.A., Boczkaj G. (2019). Integrated photocatalytic advanced oxidation system (TiO2/UV/O3/H2O2) for degradation of volatile organic compounds. Sep. Purif. Technol.

[10] Kočí K., Reli M., Troppová I., Prostějovský T., Žebrák R. (2019). Degradation of styrene from waste gas stream by advanced oxidation processes. Clean–Soil, Air, Wate.

[11] Abdolahnejad A., Mokhtari M., Ebrahimi A.A., Nikaeen M., Shahi M.A., Hajizadeh Y., to Correction (2020). Improved degradation of n-hexane vapours using a hybrid system, a photoreactor packed with TiO_2_ coated-scoria granules and a multilayer biofilter. J. Environ. Health. Eng.

[12] Fernandes A., Gągol M., Makoś P., Khan J.A., Boczkaj G. (2019). Integrated photocatalytic advanced oxidation system (TiO_2_/UV/O_3_/H_2_O_2_) for degradation of volatile organic compounds. Sep. Purif. Technol..

[13] Ji J., Xu Y., Huang H., He M., Liu S., Liu G., Xie R., Feng Q., Shu Y., Zhan Y., Fang R. (2017). Mesoporous TiO_2_ under VUV irradiation: enhanced photocatalytic oxidation for VOCs degradation at room temperature. Chem. Eng. J..

